# Assessing medium constituents for optimal heterologous production of anhydromevalonolactone in recombinant *Aspergillus oryzae*

**DOI:** 10.1186/s13568-014-0052-9

**Published:** 2014-06-27

**Authors:** Songsak Wattanachaisaereekul, Anuwat Tachaleat, Juntira Punya, Rachada Haritakun, Chollaratt Boonlarppradab, Supapon Cheevadhanarak

**Affiliations:** 1 Pilot Plant Development and Training Institute, King Mongkut’s University of Technology Thonburi, 49 Soi Thianthale 25, Bangkhunthian-Chaithale Rd., Thakham, Bangkok 10150, Bangkhunthian, Thailand; 2 Bioresource Research Unit, National Center for Genetic Engineering and Biotechnology, 113 Thailand Science Park, Phahonyothin Rd., Khlong Nueng, Klong Luang 12120, Pathum Thani, Thailand; 3 School of Bioresources and Technology, King Mongkut’s University of Technology Thonburi, 49 Soi Thianthale 25, Bangkhunthian-Chaithale Rd., Thakham, Bangkok 10150, Bangkhunthian, Thailand

**Keywords:** Anhydromevalonolactone, Aspergillus oryzae, Plackett-Burman design, Central composite design, Response surface methodology

## Abstract

Anhydromevalonolactone (AMVL) is a bioactive natural product that arises from a molecular biology technique using *Aspergillus oryzae* as a heterologous host. AMVL has been used as a precursor for the synthesis of insect pest control reagents and has numerous applications in the biotechnological and medical industries. In this study, the Plackett-Burman Design and the Central Composite Design, which offer efficient and feasible approaches, were complemented to screen significant parameters and identify the optimal values for maximum AMVL production. The results suggested that sucrose, NaNO_3_, yeast extract and K_2_HPO_4_ were the key factors affecting AMVL production in a complex medium, whereas the major components required for a defined medium were NaNO_3_, K_2_HPO_4_, KH_2_PO_4_ and trace elements. These factors were subsequently optimized using the response surface methodology. Under optimal conditions, a maximum AMVL production of 250 mg/L in the complex medium and 200 mg/L in the defined medium was achieved, which represents an increase of approximately 3-4-fold compared to the commonly used malt extract medium.

## Introduction

**Figure 1:**
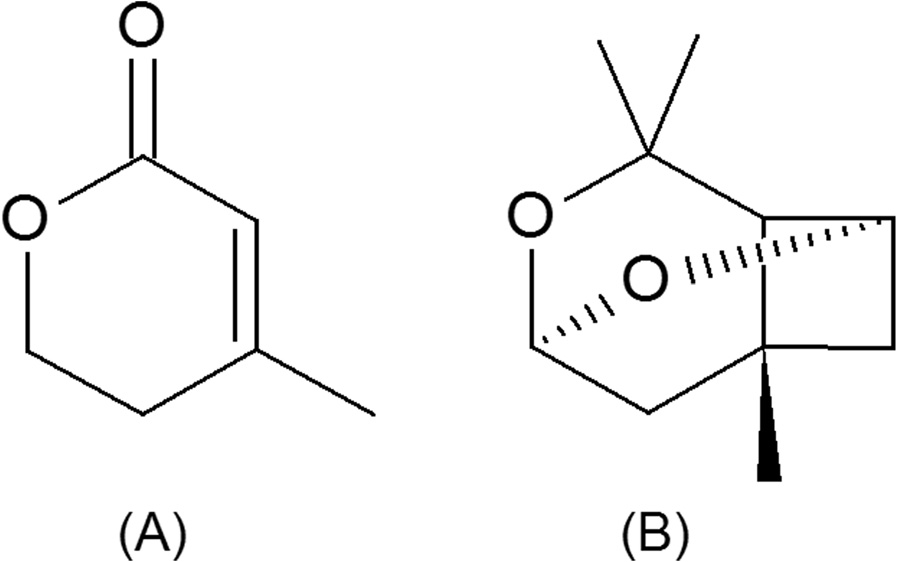
Structures of anhydromevalonolactone (A) and lineatin (B).

*Aspergillus oryzae* is an asexual, ascomycete filamentous fungus that plays an essential role in the production of oriental fermented foods and beverages such as soy sauce, miso, sake, and rice vinegar (Yokotsuka [1961]; Yong and Wood [1974]; Sakaguchi et al. [1992]). This fungus is generally regarded as safe (GRAS) by the U.S. Food and Drug Administration (FDA) and the U.S. Environmental Protection Agency because it does not appear to be pathogenic for plants or animals (U.S. Environmental Protection Agency [1997]). *A. oryzae* has long been used for large-scale industrial enzyme production: e.g., amylases, proteases, lipases and other hydrolytic enzymes (Ohnishi et al. [1994]; Carlsen and Nielsen [2001]; Chutmanop et al. [2008]). More recently, it has been used as a heterologous host for the production of anhydromevalonolactone (AMVL) (Figure 1A) and other novel pyrones based on ectopic expression of the gene encoding polyketide synthase (*pksmt*) from *Xylaria* sp. BCC 1067 under the control of the *gpdA* promoter (Punya et al. [2013]).

AMVL has been used as a precursor for the synthesis of lineatin (Figure 1B), which is a pheromone produced by the female striped ambrosia beetle (McKay et al. [1982]). This type of beetle is responsible for severe economic damage to the timber and wood industries around the world (Orbay et al. [1994]; Bumrungsri et al. [2008]). Lineatin can act as a lure for mass trapping of the ambrosia beetle; therefore, AMVL is being studied as a possible precursor for a pest control reagent. Interestingly, it has been reported that AMVL can inhibit the growth of *Lactobacillus acidophilus* and *L. heterohiochii* in the presence of mevalonic acid, and it completely inhibits the growth of *Saccharomyces cerevisiae* and *S. carlsbergensis* (Kitamura et al. [1976]). This finding indicates that AMVL can also be used as a compound to inhibit the incorporation of mevalonic acid into cholesterol in the mevalonate biosynthetic pathway. Furthermore, not only was AMVL found to be a precursor for two novel pyrones that exhibited anti-oral cavity cancer activity, but its derivatives also offer a wide range of biological activities (Punya et al. [2013]; Busch and Hertweck [2009], Wilk et al. [2009]). Therefore, AMVL has attracted a great deal of attention as a possible precursor for drugs and other valuable compounds that are of economic importance. Despite the interesting aspects of AMVL, scientific reports on its production are very limited. AMVL has previously been chemically synthesized and identified as a metabolite present in the culture broths of various fungi, but the production of AMVL has been hampered by low and unstable yields.

Malt extract broth (MEB) is the commonly used medium in our laboratory for fungal cultivation. MEB contains 17 g/L malt extract and 3 g/L mycological peptone. Malt extract is a diastase-free extract of malt and provides both nitrogenous and carbohydrate constituents; therefore, the use of this medium has been recommended for the cultivation of various molds and yeast. Because MEB was successfully used in our laboratory for the production of xyrrolin from *Xylaria* sp. BCC 1067 (phonghanpot et al. [2012]), it was further used for the production of AMVL by *Aspergillus oryzae* MTG4. Unfortunately, a low AMVL yield was obtained using this medium. Additionally, there is no report on the statistical optimization of the nutritive conditions on AMVL production. For this reason, improvement of AMVL production using culture medium optimization is required.

Generally, the experimental procedure of medium optimization is carried out by varying a single factor to find the optimal solution while other factors are kept at a constant level, and this approach is referred to as one-factor-at-a-time. This technique obviously has serious disadvantages because interactions among the factors are not considered; therefore, it does not reflect all the potential effects on the optimization process (Lundstedt et al. [1998]). More efficient approaches to determining the optimal conditions are based on the Plackett-Burman Design (PBD) and the Response Surface Methodology (RSM). The PBD is a method used to identify the components in a culture medium that have significant influences on the targeted response. Following the PBD, RSM is usually used for further optimization to detect the optimal concentration of the key components obtained from the PBD. RSM is a statistical technique for designing experiments, evaluating the relationships between independent variables and responses, searching for the optimum conditions and generating mathematical models that accurately describe the overall process (Myers [2002]). This statistical optimization method has been used extensively for optimization in many areas of industrial research and process development in chemistry and biotechnology (Khamduang et al. [2009]; Chen et al. [2010]; Guo et al. [2010]). The most popular statistical designs for RSM are the Central Composite Design (CCD) and the Box-Behnken Design (BBD). These designs differ based on the location of the experimental points in the studied region and the levels of one numeric factor (Bezerra et al. [2008]).

The present study aims to optimize the medium components of complex and defined media to improve AMVL production by the recombinant *A. oryzae*. In this study, the significant medium components were identified by PBD, and the determining factors were optimized through CCD. Finally, the optimal conditions were experimentally validated, and AMVL production with optimized culture media was compared with the initial conditions. To our best knowledge, this is the first report illustrating a statistical medium optimization process for increased AMVL production in *A. oryzae*.

## Materials and methods

### Microorganisms and culture conditions

*A. oryzae* MTG4 (BIOTEC Molecular Genomics Collection, BMGC, no. 115) (Punya et al. [2013]) harboring the gene encoding pksmt from *Xylaria* sp. BCC 1067 was cultivated on malt extract agar containing 20 g/L malt extract and 15 g/L agar, at 30°C for 1 week. The spores were harvested in 0.05% Tween 80 solution. The spores were transferred to 250-mL Erlenmeyer flasks containing 50 mL medium with different medium compositions designed by PBD and CCD. The pH of the medium was adjusted to 6.5 with 2 M HCl prior to autoclaving. With the initial spore concentration of 10^7^ spores/50 mL medium, the shake-flask cultures were incubated at 25°C on a rotary shaker at 200 rpm for 23 days. All experiments were carried out in triplicate.

### Biomass measurement and analytical methods for AMVL and sucrose

Biomass measurements were made by measuring the dry weight of biomass samples filtered with Whatman™ no. 1 filter paper (GE healthcare UK Limited) at 105°C for 48 hours. The fermentation broth was filtered through a 0.2-μm pore-sized nylon filter (VertiClean™, Vertical Chromatography Co., Ltd.) for later analysis of AMVL and sucrose. AMVL was analyzed by HPLC (Agilent Technologies 1200 series) on a device equipped with a UV detector (220 nm) and a C18 reversed-phase column (VertiSep™, UPS 4.6 × 250 mm, 5 μm, Vertical Chromatography Co., Ltd.), which was operated using a gradient of deionized water and acetonitrile at a flow rate of 0.5 mL/min. Sucrose was quantified by HPLC, which was operated at 60°C, with the flow rate of 0.6 mL/min for 5 mM H_2_SO_4_, using a refractive index detector and an ion exclusion column (Aminex HPX-87H, 300 mm × 7.8 mm, Bio-Rad Laboratories).

### Experimental designs and optimization

The approach for medium optimization adopted for improving AMVL production was divided into the following 3 steps:

### Screening of carbon and nitrogen sources

Because secondary metabolite production in filamentous fungi is strongly influenced by the carbon and nitrogen sources, the screening of carbon and nitrogen sources is crucial. A selection of carbon and nitrogen sources was therefore examined through a traditional non-statistical method. In this study, glucose, sucrose, and soluble starch were used to optimize the carbon source, and NH_4_Cl, NH_4_NO_3_, (NH_4_)_2_SO_4_, NaNO_3_, KNO_3_, urea, yeast extract and malt extract were used to optimize the nitrogen source. Based on Czapex Yeast broth (CYB), the concentrations of the carbon and nitrogen sources were set at 40 g/L and 5 g/L, respectively, and the rest of the medium components were 5 g/L K_2_HPO_4_, 0.5 g/L MgSO_4_.7H_2_O, 0.5 g/L KCl, and 10 mL/L trace elements (0.3575 g/L ZnSO_4_.7H_2_O, 0.0625 g/L CuSO_4_.5H_2_O, 0.25 g/L MnSO_4_.H_2_O, 0.345 g/L FeSO_4_.7H_2_O, and 0.075 g/L citric acid). *A. oryzae* MTG4 was cultivated in the media with different carbon and nitrogen sources on a rotary shaker at 25°C, 200 rpm for 23 days, and the fermentation broth was analyzed for AMVL production.

### Plackett-Burman design

The Plackett-Burman design has been widely and successfully used in the screening of the major constituents of cultivation media. This experimental design was adopted in this study to identify the medium components that have significant effects on AMVL production. This technique is based on the first-polynomial model according to Eq. (1):

(1) $$Y=\beta_{0}+\sum \beta_{i}x_{i}$$

where *Y* is the response, which is the concentration of AMVL, *β*_*0*_ is the model intercept, *β*_*i*_ is the linear coefficient, and *x*_*i*_ is the level of independent variables. This model does not describe the interaction among factors, but it is used to evaluate and screen the factors that significantly impact the response (*Y*). The independent variables chosen to be screened for the complex medium were sucrose, NaNO_3_, yeast extract, K_2_HPO_4,_ KH_2_PO_4_, KCl, MgSO_4_.7H_2_O and trace elements, while the nutrients selected for the defined medium were sucrose, NaNO_3_, K_2_HPO_4_, KH_2_PO_4_, KCl, MgSO_4_.7H_2_O, CaCl_2_.2H_2_O, Na_3_C_6_H_5_O_7_.2H_2_O, biotin and trace elements.

Based on the Plackett-Burman design, each factor was illustrated in two levels: −1 for the low level and +1 for the high level. A center point was run to evaluate the linear and curvature effects of the variables (Plackett and Burman, [1946]). The experimental design for screening the significant variables in AMVL production with complex and defined media are provided in Table 1. However, the concentration levels in the defined medium were defined based on the optimum concentration of each component in the complex medium.

**Table 1:** The levels of independent variables in the Plackett-Burman Design for the complex and defined media

**Varible**	**Components**	**Levels of variable**		
		**−1 (Low)**	**0 (Central)**	**+1 (High)**
	*Complex medium*			
A	Sucrose (g/L)	20	40	60
B	NaNO_3_ (g/L)	1	3	5
C	Yeast extract (g/L)	0	2	4
D	KH_2_PO_4_ (g/L)	0	1	2
E	K_2_HPO_4_ (g/L)	1	3	5
F	KCl (g/L)	0.5	0.75	1
G	MgSO_4_.7H_2_O (g/L)	0.5	0.75	1
H	Trace elements (mL/L)	0.5	0.75	1
	*Defined medium*			
I	Sucrose (g/L)	40	70	100
J	NaNO_3_ (g/L)	3	5	7
K	K_2_HPO_4_ (g/L)	3	5	7
L	KH_2_PO_4_ (g/L)	0	1	2
M	KCl (g/L)	0.25	0.50	0.75
N	MgSO_4_.7H_2_O (g/L)	0.25	0.50	0.75
O	CaCl_2_.2H_2_O (g/L)	0	0.10	0.20
P	Na_3_C_6_H_5_O_7_.2H_2_O (g/L)	0	1	2
Q	Biotin (mL/L)	0	0.10	0.20
R	Trace elements (mL/L)	0.25	0.50	0.75

In this study, the variables were screened in 12 experimental runs with the foldover augmenting method to increase the resolution of the design in addition to 6 runs at the center points, giving a total of 30 experimental runs. All experiments were performed in triplicate, and the average concentration of AMVL was used as the response (the dependent variable). The factors significant at 95% (p < 0.05) were considered to have a significant effect on AMVL production and were used for further optimization by the response surface methodology.

### Central composite design

Response surface methodology was carried out to illustrate the nature of the response surface in the experimental region and to optimize the medium components for enhanced AMVL production by using the central composite design. For the complex medium, 4 critical components identified based on the Plackett-Burman design, namely, sucrose, NaNO_3_, yeast extract and K_2_HPO_4,_ were optimized, whereas NaNO_3_, K_2_HPO_4_, KH_2_PO_4_ and trace elements were optimized for the defined medium.

According to the experimental design, a full 2^4^ factorial design, 8 axial points and 6 replications of center points were used, which led to 30 sets of experiments. Each factor optimized for the complex and defined media was studied at five different levels (−2, −1, 0, +1, +2). Other non-significant factors were held constant. The relationship between independent variables and responses was described by a second order polynomial equation, Eq. (2):

(2) $$Y=\beta_{0}+\sum \beta_{i}x_{i}+\sum \beta_{\mathrm{ii}}{x_{i}}^{2}+\sum \beta_{\mathrm{ij}}x_{i}x_{j},\;i=1,2,\ldots k$$

where *Y* is the predicted response, *x*_*i*_ and *x*_*j*_ are the independent variables that influence the response *Y, β*_*0*_ is the offset term, *β*_*i*_ is the linear coefficient of *x*_*i*_, *β*_*ii*_ is the quadratic coefficient of *x*_*i*_, and *β*_*ij*_ is the interaction coefficient between *x*_*i*_ and *x*_*j*_. All experiments were carried out in triplicate. The results were analyzed and interpreted using Design Expert Version 8 (Design-Expert®, Stat-Ease, Inc. Minneapolis, MN, USA). The statistical significance of the second-order model was determined using Fischer’s test, and the significances of all terms in the polynomial were assessed according to their p-values. The quality of the regression model was assessed statistically by the coefficient of determination R^2^. Response surface plots were generated, and the optimum concentration of each variable was calculated by the differential equation of the quadratic model. Finally, an experiment on the optimal medium was conducted to validate the central composite design model developed.

## Results

### Screening of optimal carbon and nitrogen sources

To choose the most efficient nutrients, glucose, sucrose, and soluble starch were used as carbon sources, and various compounds containing nitrogen, namely NH_4_Cl, NH_4_NO_3_, (NH_4_)_2_SO_4_, NaNO_3_, KNO_3_, urea, yeast extract and malt extract, were used as nitrogen sources for AMVL production by *A. oryzae* MTG4. Based on CYB medium, the concentrations of carbon and nitrogen sources were initially set as 40 g/L and 5 g/L, respectively, in addition to 5 g/L K_2_HPO_4_, 0.5 g/L KCl, 0.5 g/L MgSO_4_.7H_2_O and 10 mL/L trace elements.

**Figure 2:**
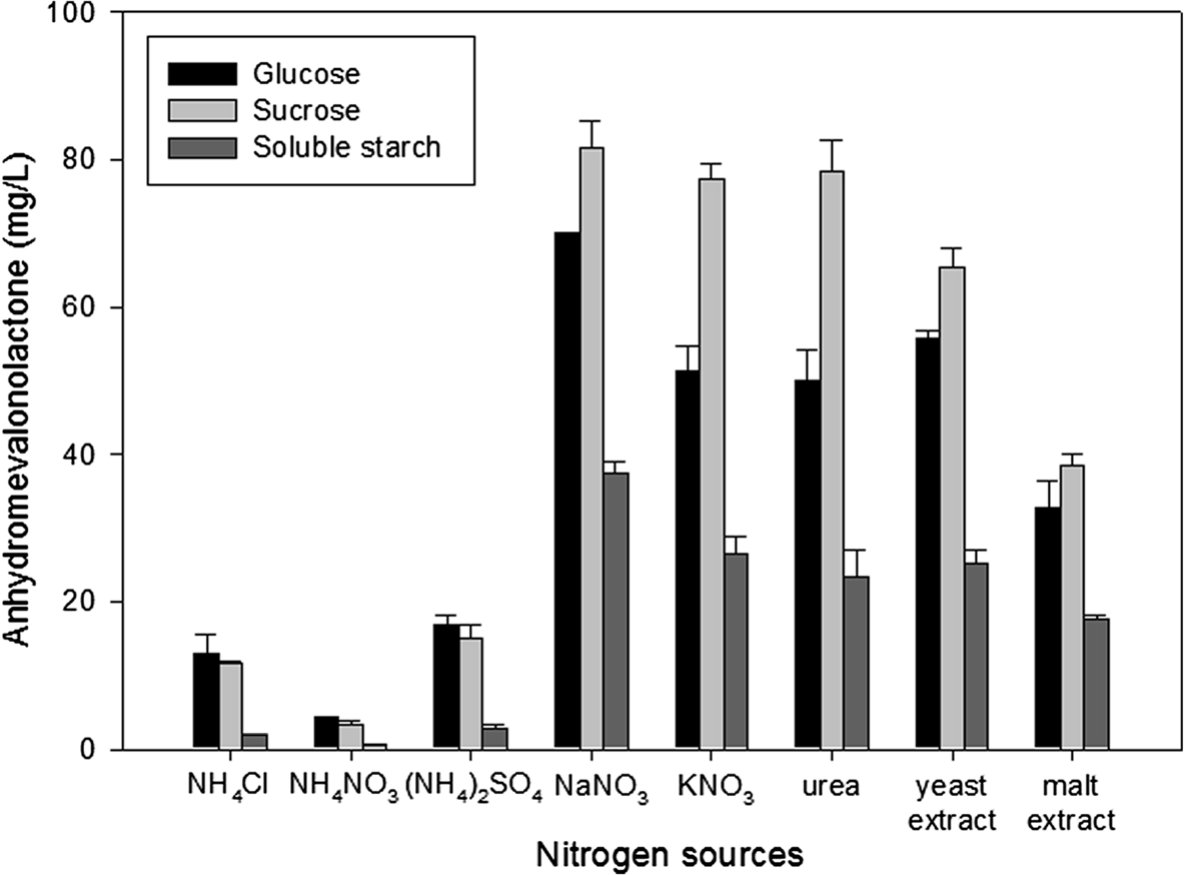
Effects of different carbon and nitrogen sources on AMVL production.

As illustrated in Figure 2, AMVL production varied with the different carbon and nitrogen sources. Sucrose, which provides an energy source, was a more suitable carbon source for AMVL production than glucose and soluble starch, while NaNO_3_ was the most preferable nitrogen source. The combined use of sucrose and NaNO_3_ led to the highest AMVL production at 82 mg/L. Yeast extract, which is an extract of autolyzed brewer’s yeast and provides essential nutrients such as vitamins, amino acids and other nitrogenous compounds required for fungal growth, is also beneficial for AMVL production. AMVL produced approximately 65 mg/L with the combination of sucrose and yeast extract. Additionally, it was observed that AMVL production was low in the media containing ammonium ions such as NH_4_Cl, NH_4_NO_3_ and (NH_4_)_2_SO_4_ because after the ammonium ions were used up, the media became acidic and unfavorable for AMVL production. Therefore, in this study, sucrose and NaNO_3_ were selected as carbon and inorganic nitrogen sources for the defined medium, respectively, whereas yeast extract were chosen as an organic nitrogen source for the complex medium.

### Plackett-Burman design

The Plackett-Burman design is the screening design that was used to identify important variables that had significant effects on AMVL production. In this experimental design, the exact quantity of each variable is not determined, and the interactions between the variables are considered negligible (Plackett and Burman [1946]). The design matrix and its corresponding AMVL concentration with the complex and defined media are illustrated in Tables 2 and 3, respectively.

**Table 2:** Experimental design using the Plackett-Burman method with AMVL production for the complex medium

**Run**	**Coded values**								**AMVL (mg/L)**
	**A**	**B**	**C**	**D**	**E**	**F**	**G**	**H**	
1	1	1	−1	1	1	1	−1	−1	161.1 ± 7.2
2	−1	1	1	−1	1	1	1	−1	139.1 ± 5.3
3	1	−1	1	1	−1	1	1	1	17.6 ± 0.3
4	−1	1	−1	1	1	−1	1	1	68.3 ± 4.7
5	−1	−1	1	−1	1	1	−1	1	50.7 ± 3.1
6	−1	−1	−1	1	−1	1	1	−1	1.4 ± 0.1
7	1	−1	−1	−1	1	−1	1	1	12.4 ± 0.5
8	1	1	−1	−1	−1	1	−1	1	81.0 ± 3.6
9	1	1	1	−1	−1	−1	1	−1	123.8 ± 5.8
10	−1	1	1	1	−1	−1	−1	1	55.0 ± 3.2
11	1	−1	1	1	1	−1	−1	−1	39.2 ± 1.6
12	−1	−1	−1	−1	−1	−1	−1	−1	1.1 ± 0.1
13	0	0	0	0	0	0	0	0	80.5 ± 4.4
14	0	0	0	0	0	0	0	0	86.2 ± 4.7
15	0	0	0	0	0	0	0	0	81.2 ± 3.5
16	−1	−1	1	−1	−1	−1	1	1	34.4 ± 2.0
17	1	−1	−1	1	−1	−1	−1	1	1.6 ± 0.2
18	−1	1	−1	−1	1	−1	−1	−1	74.8 ± 3.4
19	1	−1	1	−1	−1	1	−1	−1	48.1 ± 2.2
20	1	1	−1	1	−1	−1	1	−1	86.0 ± 4.1
21	1	1	1	−1	1	−1	−1	1	136.1 ± 5.7
22	−1	1	1	1	−1	1	−1	−1	80.9 ± 3.8
23	−1	−1	1	1	1	−1	1	−1	57.2 ± 2.3
24	−1	−1	−1	1	1	1	−1	1	2.4 ± 0.3
25	1	−1	−1	−1	1	1	1	−1	25.0 ± 0.9
26	−1	1	−1	−1	−1	1	1	1	44.5 ± 2.3
27	1	1	1	1	1	1	1	1	142.9 ± 6.8
28	0	0	0	0	0	0	0	0	92.8 ± 3.7
29	0	0	0	0	0	0	0	0	76.9 ± 2.9
30	0	0	0	0	0	0	0	0	87.1 ± 3.3

Note: A, Sucrose; B, NaNO_3_; C, Yeast extract; D, KH_2_PO_4_; E, K_2_HPO_4_; F, KCl; G, MgSO_4_.7H_2_O; H, Trace elements.

**Table 3:** Experimental design using the Plackett-Burman method with AMVL production for the defined medium

**Run**	**Coded values**										**AMVL (mg/L)**
	**I**	**J**	**K**	**L**	**M**	**N**	**O**	**P**	**Q**	**R**	
1	1	1	−1	1	1	1	−1	−1	−1	1	44.5 ± 2.2
2	−1	1	1	−1	1	1	1	−1	−1	−1	57.9 ± 3.1
3	1	−1	1	1	−1	1	1	1	−1	−1	32.6 ± 1.4
4	−1	1	−1	1	1	−1	1	1	1	−1	54.7 ± 2.6
5	−1	−1	1	−1	1	1	−1	1	1	1	17.7 ± 0.6
6	−1	−1	−1	1	−1	1	1	−1	1	1	11.7 ± 0.4
7	1	−1	−1	−1	1	−1	1	1	−1	1	24.2 ± 1.3
8	1	1	−1	−1	−1	1	−1	1	1	−1	89.9 ± 3.8
9	1	1	1	−1	−1	−1	1	−1	1	1	38.4 ± 1.5
10	−1	1	1	1	−1	−1	−1	1	−1	1	23.8 ± 1.1
11	1	−1	1	1	1	−1	−1	−1	1	−1	19.5 ± 0.8
12	−1	−1	−1	−1	−1	−1	−1	−1	−1	−1	56.2 ± 3.3
13	0	0	0	0	0	0	0	0	0	0	45.4 ± 1.7
14	0	0	0	0	0	0	0	0	0	0	41.8 ± 1.9
15	0	0	0	0	0	0	0	0	0	0	39.9 ± 1.6
16	−1	−1	1	−1	−1	−1	1	1	1	−1	41.5 ± 2.3
17	1	−1	−1	1	−1	−1	−1	1	1	1	20.1 ± 0.7
18	−1	1	−1	−1	1	−1	−1	−1	1	1	56.4 ± 3.0
19	1	−1	1	−1	−1	1	−1	−1	−1	1	21.8 ± 1.1
20	1	1	−1	1	−1	−1	1	−1	−1	−1	58.4 ± 2.9
21	1	1	1	−1	1	−1	−1	1	−1	−1	71.4 ± 3.7
22	−1	1	1	1	−1	1	−1	−1	1	−1	71.8 ± 4.3
23	−1	−1	1	1	1	−1	1	−1	−1	1	8.8 ± 0.2
24	−1	−1	−1	1	1	1	−1	1	−1	−1	45.3 ± 1.9
25	1	−1	−1	−1	1	1	1	−1	1	−1	42.0 ± 2.2
26	−1	1	−1	−1	−1	1	1	1	−1	1	52.1 ± 3.5
27	1	1	1	1	1	1	1	1	1	1	47.5 ± 2.4
28	0	0	0	0	0	0	0	0	0	0	47.0 ± 3.2
29	0	0	0	0	0	0	0	0	0	0	41.6 ± 1.8
30	0	0	0	0	0	0	0	0	0	0	40.5 ± 1.5

Note: I, Sucrose; J, NaNO_3_; K, K_2_HPO_4_; L, KH_2_PO_4_; M, KCl; N, MgSO_4_.7H_2_O; O, CaCl_2_.2H_2_O; P, Na_3_C_6_H_5_O_7_.2H_2_O; Q, Biotin; R, Trace elements.

For the complex medium, the p-value, which indicates the statistical confidence of an estimated factor, and the main effects of each medium constituent are summarized in Table 4. According to the statistical analysis, sucrose, NaNO_3_, yeast extract, K_2_HPO_4_, KCl and MgSO_4_.7H_2_O had positive effects. However, KH_2_PO_4_ and trace elements showed negative effects on AMVL production. A maximum p-value of 0.05 was used as a cutoff point for selecting significant variables. The result suggested that only four variables, sucrose, NaNO_3_, yeast extract and K_2_HPO_4_, were significant factors because their p-values were 0.0201, <0.001, 0.0025 and 0.0049, respectively. Because these factors showed positive effects as previously mentioned, a +1 level would be expected to improve AMVL production. Other variables had low confidence levels because the p-values were greater than 0.05, and consequently, these variables were considered insignificant and were not included in the next optimization experiment.

**Table 4:** Statistical analysis of the Plackett-Burman design for the complex and defined media

**Variable**	**Effect**	**Coefficient**	**p-value**
*Complex medium*			
constant		65.04	< 0.0001
A, Sucrose	22.1004	11.05	0.0201
B, NaNO_3_	75.1828	37.59	< 0.0001
C, Yeast extract	30.4603	15.23	0.0025
D, KH_2_PO_4_	−4.7852	−2.39	0.5877
E, K_2_HPO_4_	27.8186	13.91	0.0049
F, KCl	8.7311	4.37	0.3272
G, MgSO_4_.7H_2_O	1.7222	0.86	0.8448
H, Trace elements	−15.9140	−7.96	0.0830
*Defined medium*			
constant		42.14	< 0.0001
I, Sucrose	1.0233	0.51	0.7089
J, NaNO_3_	27.1197	13.56	< 0.0001
K, K_2_HPO_4_	−8.5673	−4.28	0.0052
L, KH_2_PO_4_	−10.8947	−5.45	0.0008
M, KCl	−2.3609	−1.18	0.3930
N, MgSO_4_.7H_2_O	5.1297	2.56	0.0733
O, CaCl_2_.2H_2_O	−5.7316	−2.87	0.0542
P, Na_3_C_6_H_5_O_7_.2H_2_O	2.7889	1.39	0.3149
Q, Biotin	1.1972	0.6	0.6625
R, Trace elements	−22.8260	−11.41	< 0.0001

For the defined medium, ten media constituents were screened by PBD. The main effects, including the p-values, are presented in Table 4. Based on the results, NaNO_3_, K_2_HPO_4_, KH_2_PO_4_ and trace elements had confidence levels above 95%, indicating that they significantly influenced AMVL production. Therefore, the optimal level of these factors was further determined by central composite design. The rest of the factors were considered insignificant because their confidence levels were below 95%. As a result, the insignificant factors were not included in the next round of optimization.

### Response surface methodology

The response surface methodology (RSM) is a mathematical and statistical technique used for developing and optimizing processes. This technique is also used for evaluating the significance of relevant factors in experiments. Central composite design (CCD), which is a popular experimental design in RSM, has been successfully used for medium optimization. CCD has three sets of experimental runs as follows: (1) full factorial runs in which each factor is studied at +1 and −1 levels, (2) replication at the center points of all factors to help in understanding the curvature and to estimate pure error, and (3) axial points with the values outside the median of two factorial levels. The factors optimized for the complex and defined media are listed in Table 5.

**Table 5:** Coded and uncoded values of independent variables used in the central composite design for the complex and defined media

**Independent variables**	**Coded value**				
	**-α(−2)**	**−1**	**0**	**+1**	**+α(+2)**
*Complex medium*					
A, Sucrose (g/L)	10	40	70	100	130
B, NaNO_3_ (g/L)	1	3	5	7	9
C, Yeast extract (g/L)	1	3	5	7	9
D, K_2_HPO_4_ (g/L)	1	3	5	7	9
*Defined medium*					
A, NaNO_3_ (g/L)	3	5	7	9	11
B, K_2_HPO_4_ (g/L)	1	2	3	4	5
C, KH_2_PO_4_ (g/L)	0	0.5	1	1.5	2
D, Trace elements (mL/L)	0	0.15	0.30	0.45	0.60

### Optimization of the complex medium for AMVL production

According to the screening results obtained by the Plackett-Burman design, sucrose, NaNO_3_, yeast extract and K_2_HPO_4_ were chosen as independent input variables, while AMVL production was used as an output variable. A 2^4^-factorial central composite experimental design, with six replications at the center points and a 2 × 4 axial point (α = 2) leading to a total number of 30 experimental runs, was employed for the optimization. Table 6 shows the central composite design along with the predicted and observed response for each individual experiment. The relationships between sucrose, NaNO_3_, yeast extract and K_2_HPO_4_ were identified by RSM. The analysis of variance (ANOVA) for the quadratic response surface model is presented in Table 7, and the full second-order polynomial equation for AMVL production was given in coded values as Eq. (3):

(3) $$Y=242.18+3.80A+7.53B+5.31C+17.19D+7.96\mathrm{AB}+1.81\mathrm{AC}-5.76\mathrm{AD}-11.90\mathrm{BC}-0.28\mathrm{BD}\;-6.16\mathrm{CD}-15.62A^{2}-19.60B^{2}-14.73C^{2}-13.41D^{2}$$

where *Y* is the predicted AMVL production (mg/L), A is sucrose, B is NaNO_3_, C is yeast extract, D is K_2_HPO_4_, and AB represents the interaction term between A and B.

**Table 6:** Central composite design for the complex medium

**Run**	**Coded factors**				**AMVL (mg/L)**	
	**A**	**B**	**C**	**D**	**Actual**	**Predicted**
1	−1	−1	−1	−1	137.9 ± 5.3	129.1
2	1	−1	−1	−1	134.5 ± 6.2	132.3
3	−1	1	−1	−1	157.9 ± 7.1	152.1
4	1	1	−1	−1	183.0 ± 7.7	187.1
5	−1	−1	1	−1	176.8 ± 8.4	175.9
6	1	−1	1	−1	184.9 ± 8.0	179.1
7	−1	1	1	−1	154.0 ± 6.5	151.2
8	1	1	1	−1	189.8 ± 8.3	186.3
9	−1	−1	−1	1	195.0 ± 8.6	187.4
10	1	−1	−1	1	165.8 ± 7.5	167.5
11	−1	1	−1	1	211.6 ± 9.7	210.3
12	1	1	−1	1	224.1 ± 9.3	222.3
13	−1	−1	1	1	214.6 ± 9.1	209.5
14	1	−1	1	1	194.9 ± 6.8	189.6
15	−1	1	1	1	185.4 ± 5.7	184.8
16	1	1	1	1	195.2 ± 7.9	196.8
17	−2	0	0	0	161.0 ± 7.6	172.1
18	2	0	0	0	187.2 ± 6.4	187.3
19	0	−2	0	0	137.2 ± 4.1	148.7
20	0	2	0	0	179.2 ± 6.8	178.8
21	0	0	−2	0	167.3 ± 6.7	172.6
22	0	0	2	0	188.1 ± 7.3	193.9
23	0	0	0	−2	146.8 ± 5.2	154.2
24	0	0	0	2	219.2 ± 8.9	222.9
25	0	0	0	0	238.9 ± 9.5	242.2
26	0	0	0	0	248.8 ± 8.0	242.2
27	0	0	0	0	241.5 ± 8.2	242.2
28	0	0	0	0	243.3 ± 9.4	242.2
29	0	0	0	0	233.1 ± 6.7	242.2
30	0	0	0	0	247.5 ± 7.2	242.2

Note: A, Sucrose; B, NaNO_3_; C, Yeast extract; D, K_2_HPO_4_.

**Table 7:** ANOVA for response surface quadratic model of the complex and defined media

	**Sum of**		**Mean**	**F**	**p-value**	
**Source**	**Squares**	**df**	**Square**	**Value**	**Prob > F**	
*Complex medium*						
Model	33907.83	14	2421.99	44.71	< 0.0001	Significant
A-Sucrose	345.73	1	345.73	6.38	0.0233	
B-NaNO_3_	1359.75	1	1359.75	25.1	0.0002	
C-Yeast extract	675.49	1	675.49	12.47	0.003	
D-K_2_HPO_4_	7094.29	1	7094.29	130.97	< 0.0001	
AB	1015	1	1015	18.74	0.0006	
AC	52.56	1	52.56	0.97	0.3402	
AD	530.92	1	530.92	9.8	0.0069	
BC	2264.16	1	2264.16	41.8	< 0.0001	
BD	1.27	1	1.27	0.023	0.8806	
CD	606.84	1	606.84	11.2	0.0044	
A^2^	6695.69	1	6695.69	123.61	< 0.0001	
B^2^	10541.5	1	10541.5	194.61	< 0.0001	
C^2^	5955.23	1	5955.23	109.94	< 0.0001	
D^2^	4934.49	1	4934.49	91.1	< 0.0001	
Residual	812.53	15	54.17			
Lack of Fit	645.21	10	64.52	1.93	0.2428	Not significant
Pure Error	167.32	5	33.46			
Cor Total	34720.36	29				
*Defined medium*						
Model	61950.92	14	4425.07	23.27	< 0.0001	Significant
A-NaNO_3_	1466.37	1	1466.37	7.71	0.0141	
B-K_2_HPO_4_	7429.48	1	7429.48	39.07	< 0.0001	
C-KH_2_PO_4_	1619.47	1	1619.47	8.52	0.0106	
D-Trace elements	3979	1	3979	20.92	0.0004	
AB	240.85	1	240.85	1.27	0.2781	
AC	3034.41	1	3034.41	15.96	0.0012	
AD	12478.18	1	12478.18	65.61	< 0.0001	
BC	1745.83	1	1745.83	9.18	0.0084	
BD	1394.94	1	1394.94	7.33	0.0162	
CD	1498.72	1	1498.72	7.88	0.0133	
A^2^	4217.35	1	4217.35	22.18	0.0003	
B^2^	11914.43	1	11914.43	62.65	< 0.0001	
C^2^	42.42	1	42.42	0.22	0.6435	
D^2^	15571	1	15571	81.88	< 0.0001	
Residual	2852.69	15	190.18			
Lack of Fit	2166.36	10	216.64	1.58	0.3207	Not significant
Pure Error	686.33	5	137.27			
Cor Total	64803.62	29				

Note: For the complex medium: R^2^ = 0.9766, R^2^ (adj) = 0.9548, R^2^ (pred) = 0.8860, CV = 3.84%, Adeq Precision = 21.513.

For the defined medium: R^2^ = 0.956, R^2^ (adj) = 0.9149, R^2^ (pred) = 0.7922, CV = 12.93%, Adeq Precision = 17.282.

The model determination coefficient (R^2^) of the above equation was 0.9766, indicating a good agreement between the experimental and predicted values. The sample variation of 97.66% was attributed to the variable, and only less than 2.34% of the total variance could not be explained by the model. The adjusted R^2^ of 0.9548 was in reasonable agreement with the predicted R^2^ of 0.8860. The model of adequate precision (signal-to-noise ratio) of 21.513, which is very high compared to the desirable value (greater than 4), indicates an adequate signal and that the model can be used to navigate the design space. Finally, the lower value of the coefficient of variation (C.V.) was 3.84, showing that the experiments were precise and reliable (Box et al. [1978]).

To measure how well the factors describe the variation of the mean data, the F-test was used in this study. A greater F-value indicates that the factors adequately explain the variation in the data. A p-value < 0.05 indicates the significant model term. It was observed that the quadratic regression model (Eq. [3]) was highly significant because of a very low probability value (p_model_ > F = 0.0001). Additionally, the lack of fit (LOF), which is a variation of the data around the fitted model, was also used to determine the adequacy of the model fit. If the model does not fit the data well, this term is significant. As shown in Table 7, LOF was not significant at the F-value and p-value of 1.93 and 0.2428, respectively. This result indicated that the model fitted the response well and that there is a 24.28% chance that a lack of fit F-value could occur due to noise.

According to Table 7, the variables that were highly significant (P < 0.0001) were the square terms of sucrose (A^2^), NaNO_3_ (B^2^), yeast extract (C^2^), and K_2_HPO_4_ (D^2^). Moreover, the linear effect of all variables was significant. The significance of the linear and quadratic terms indicated that sucrose, NaNO_3_, yeast extract and K_2_HPO_4_ can act as limiting nutrients, and little variation in their concentrations would alter AMVL production to a considerable extent. Based on the interaction terms, the results clearly showed that the interaction terms between sucrose and yeast extract (AC) and NaNO_3_ and K_2_HPO_4_ (BD) were not significant because their p-values were higher than 0.05. Although the full model was significantly fitted to the data, it was necessary to remove those non-significant interaction terms from the full model. The polynomial model for AMVL production considered only the significant term that was expressed as a coded value by Eq. (4):

(4) $$Y=242.18+3.80A+7.53B+5.31C+17.19D+7.96\mathrm{AB}-5.76\mathrm{AD}-11.90\mathrm{BC}-6.16\mathrm{CD}-15.62A^{2}-19.60B^{2}-14.73C^{2}-13.41D^{2}$$

where *Y* is the predicted AMVL production (mg/L), A is sucrose, B is NaNO_3_, C is yeast extract, and D is K_2_HPO_4_.

**Figure 3:**
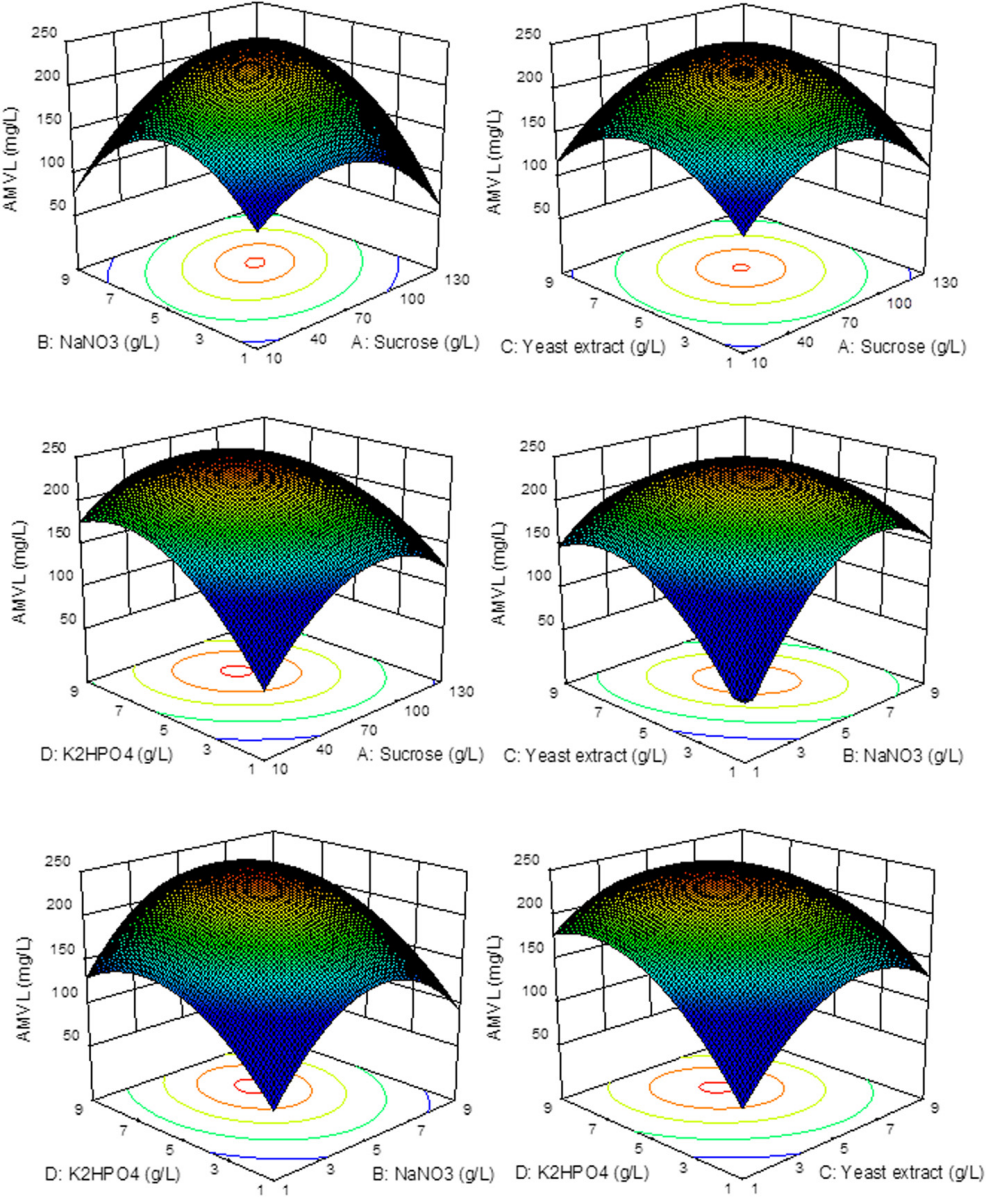
**Response surface plots of AMVL production in the complex medium as a function of sucrose (A), NaNO**_**3**_**(B), yeast extract (C) and K**_**2**_**HPO**_**4**_**(D) based on the results of the central composite design.**

According to Eq. 4, the effects of the interactions of sucrose, NaNO_3_, yeast extract and K_2_HPO_4_ on AMVL production were studied by observing the interactions of two of these variables while maintaining the other two independent variables at constant levels. The 3D response surface graphs can be used to determine the optimum values of the variables within the range considered. The 3D plots for interactions between two variables were presented in Figure 3. The result showed that an elliptical response surface was derived from the second order quadratic equation for AMVL production with the interactions between sucrose and NaNO_3_, sucrose and K_2_HPO_4_, NaNO_3_ and yeast extract, and yeast extract and K_2_HPO_4_. The predicted AMVL production decreased at the higher and lower values of these independent parameters. The maximum predicted value, identified by the surface confined in the smallest ellipse in the contour plot, was located near the center points of the response surface. The study of response surface graphs revealed that the maximum AMVL production of 248.478 mg/L was predicted with the media containing 71.651 g/L sucrose (A = 0.055), 5.418 g/L NaNO_3_ (B = 0.209), 4.931 g/L yeast extract (C = −0.0345) and 6.271 g/L K_2_HPO_4_ (D = 0.6355).

### Optimization of the defined medium for AMVL production

The defined medium, known as chemically defined medium or synthetic medium, is the medium in which all the chemicals used are known and does not contain any yeast, animal or plant tissue. The media constituents, namely, NaNO_3_, K_2_HPO_4_, KH_2_PO_4_ and trace elements as independent variables screened by PBD, were further studied by the CCD. The CCD consisted of a 2^4^ full factorial design, 2 × 4 axial designs and 6 center points. The design matrix of CCD, the independent variables and the corresponding responses are presented in Table 8.

**Table 8:** Central composite design for the defined medium

**Run**	**Coded factors**				**AMVL (mg/L)**	
	**A**	**B**	**C**	**D**	**Actual**	**Predicted**
1	−1	−1	−1	−1	157.6 ± 7.6	143.0
2	1	−1	−1	−1	141.9 ± 6.3	130.3
3	−1	1	−1	−1	71.0 ± 3.7	68.2
4	1	1	−1	−1	43.0 ± 2.4	55.6
5	−1	−1	1	−1	117.3 ± 5.9	113.9
6	1	−1	1	−1	49.1 ± 2.2	46.1
7	−1	1	1	−1	81.2 ± 3.5	80.9
8	1	1	1	−1	21.8 ± 2.6	13.1
9	−1	−1	−1	1	68.2 ± 3.2	74.9
10	1	−1	−1	1	177.4 ± 9.1	173.9
11	−1	1	−1	1	38.2 ± 3.3	37.5
12	1	1	−1	1	135.1 ± 7.4	136.5
13	−1	−1	1	1	85.3 ± 4.0	84.4
14	1	−1	1	1	143.3 ± 7.5	128.4
15	−1	1	1	1	94.8 ± 4.3	88.8
16	1	1	1	1	106.4 ± 5.7	132.8
17	−2	0	0	0	80.5 ± 3.6	86.1
18	2	0	0	0	122.1 ± 5.2	117.4
19	0	−2	0	0	86.0 ± 4.4	103.2
20	0	2	0	0	49.1 ± 3.5	32.8
21	0	0	−2	0	171.9 ± 8.6	167.8
22	0	0	2	0	139.9 ± 7.4	134.9
23	0	0	0	−2	19.8 ± 2.6	30.3
24	0	0	0	2	91.4 ± 5.8	81.8
25	0	0	0	0	141.9 ± 7.4	151.3
26	0	0	0	0	135.8 ± 6.1	151.3
27	0	0	0	0	154.2 ± 7.3	151.3
28	0	0	0	0	168.5 ± 8.7	151.3
29	0	0	0	0	149.4 ± 7.0	151.3
30	0	0	0	0	158.3 ± 8.2	151.3

Note: A, NaNO_3_; B, K_2_HPO_4_; C, KH_2_PO_4_; D, Trace elements.

To determine the optimal value, the ANOVA for the full second-order polynomial quadratic model was carried out as shown in Table 7. By applying multiple regression analysis to the experimental data, the second-order polynomial equation was developed as a coded value as Eq. (5):

(5) $$Y=151.33+7.82A-17.59B-8.21C+12.88D-3.88\mathrm{AB}-13.77\mathrm{AC}+27.93\mathrm{AD}+10.45\mathrm{BC}+9.34\mathrm{BD}+9.68\mathrm{CD}-12.40A^{2}-20.84B^{2}+1.24C^{2}-23.83D^{2}$$

where *Y* is the predicted AMVL production (mg/L) and A, B, C and D represent the coded values of NaNO_3_, K_2_HPO_4_, KH_2_PO_4_ and trace elements, respectively. Eq. (5) is a full model because it includes all terms regardless of their significance. The fitness of the full model was estimated by the determination coefficient (R^2^), which was 0.956, indicating a good correlation between the experimental and predicted value of AMVL production, and above 95% of the variability in the response could be explained by the mode. Additionally, the predicted determination coefficient (predicted R^2^ = 0.7922) was in reasonable agreement with the adjusted determination coefficient (adjusted R^2^ = 0.9149). The model F-values of 23.27 with p-values less than 0.0001 also implied that the model was significant. Moreover, the lack of fit for an F-value of 1.58 implied that the LOF is not significant relative to the pure error, indicating that the model fitted the data well. Although the full model showed a significant fit, there were non-significant terms (p ≥ 0.05) in the model, which were the square terms of KH_2_PO_4_ and the interaction terms between NaNO_3_ and K_2_HPO_4_. After excluding the non-significant terms, the model was modified as Eq. (6):

(6) $$Y\;=\;151.33+7.82A-17.59B-8.21C+12.88D-13.77\mathrm{AC}+27.93\mathrm{AD}+10.45\mathrm{BC}+9.34\mathrm{BD}+9.68\mathrm{CD}-12.40A^{2}-20.84B^{2}-23.83D^{2}$$

**Figure 4:**
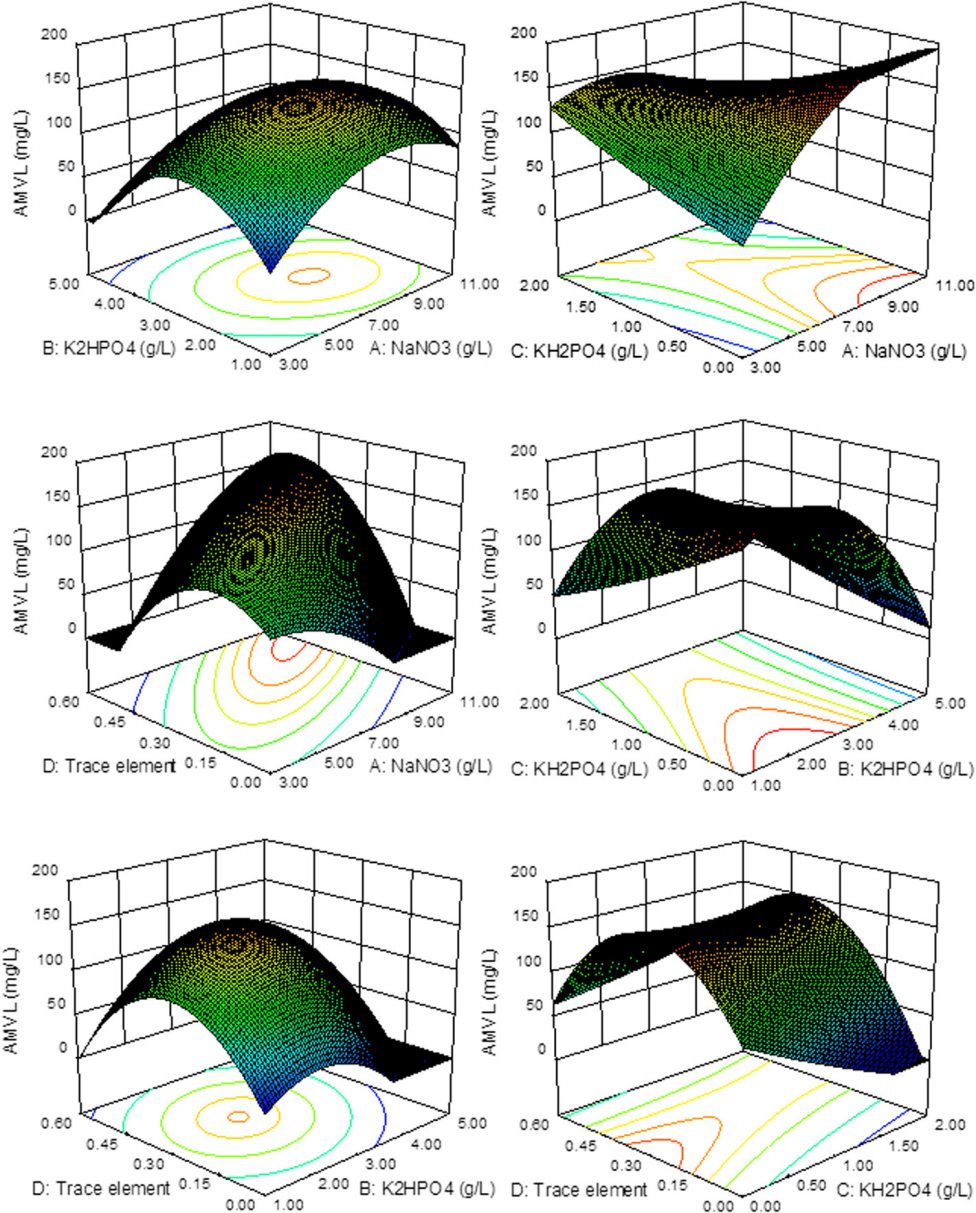
**Response surface plots of AMVL production in the defined medium as a function of NaNO**_**3**_**(A), K**_**2**_**HPO**_**4**_**(B), KH**_**2**_**PO**_**4**_**(C) and trace elements (D) based on the results of the central composite design.**

The response surface plots were used to explain the interactions of NaNO_3_, K_2_HPO_4_, KH_2_PO_4_ and trace elements as illustrated in Figure 4. Each figure showed the effect of two variables, while others were held at level zero. According to Figure 4, AMVL production was affected by varying the concentrations of NaNO_3_, K_2_HPO_4_, KH_2_PO_4_ and trace elements. The predicted AMVL production decreased at the higher and lower value ranges for both K_2_HPO_4_ and trace elements, but it increased with the increasing concentration of NaNO_3_. In contrast, the AMVL production increased with decreasing KH_2_PO_4_ concentrations because KH_2_PO_4_ showed a negative influence on AMVL production. With Eq. (6), it can be shown that the optimum point is located at A = 1.562, B = −0.568, C = −1.706 and D = 0.647 with the corresponding actual concentrations of 9.687 g/L NaNO_3_, 2.432 g/L K_2_HPO_4_, 0.147 g/L KH_2_PO_4_, and 0.397 mL/flask trace elements with a maximum predicted AMVL production of 209.85 mg/L.

### Validation of the experimental design

**Figure 5:**
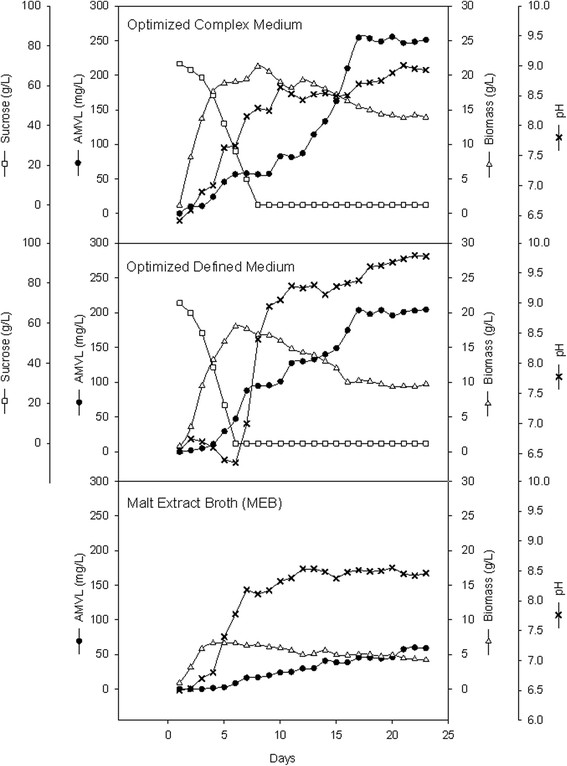
**AMVL production, changes in pH, sucrose concentration and growth curves of** ***A. oryzae*** **MTG4 in optimized media and MEB.**

According to the results obtained from the PBD and CCD, the optimized complex medium for AMVL production was prepared as follows: 71.65 g/L sucrose, 5.42 g/L NaNO_3_, 4.93 g/L yeast extract, 6.27 g/L K_2_HPO_4_, 0.5 g/L KCl, 0.5 g/L MgSO_4_.7H_2_O, and 0.25 mL/flask trace elements. In contrast, the optimal concentration of the defined medium components were 70 g/L sucrose, 9.69 g/L NaNO_3_, 2.43 K_2_HPO_4_, 0.15 g/L KH_2_PO_4_, 0.4 mL/flask trace elements, 2 g/L Na_3_C_6_H_5_O_7_.2H_2_O, 0.25 g/L KCl, and 0.75 g/L MgSO_4_.7H_2_O. The initial pH of both media was adjusted to 6.5. To validate the prediction of the model, *A. oryzae* MTG4 was characterized using two optimized media and a non-optimized medium (MEB) in triplicate. The cultivations were carried out for 23 days to investigate the influence of the media on the growth of *A. oryzae* MTG4 and AMVL production. Samples were taken for the analysis of biomass dry weight, sucrose concentration and the amount of AMVL produced. The results from cultivations using three different media are illustrated in Figure 5 and showed that exponential growth was observed in all cultivations. During this phase, the cells consumed carbon sources at their maximum rate until the carbon was exhausted. A maximum specific growth rate (μ_max_) of 0.039 h^−1^ was observed in MEB medium, whereas the μ_max_ of *A. oryzae* MTG4 in the optimized complex and defined media were 0.080 and 0.052 hr^−1^, respectively (Table 9). At the end of the cultivation, the cultures reached a phase where AMVL was constantly produced. The maximum titer of AMVL was approximately 60 mg/L in the MEB medium, whereas AMVL was approximately 200 and 250 mg/L in the optimized defined and complex media, respectively. The yields of AMVL per biomass in the optimized defined and complex media were also higher compared to the yields of AMVL in the MEB medium (Table 9). Thus, the yield and production of AMVL clearly improved when the optimal media were used for the cultivation of *A. oryzae* MTG4. However, under the calculated optimal culture conditions, the maximum AMVL concentrations produced from the defined and complex media were 209.9 and 248.5 mg/L, respectively, which agreed well with the experimental results and suggested that the models (Eq. [4] and Eq. [6]) were valid for predicting the AMVL production in the complex and defined media.

**Table 9:** **Maximum specific growth rate, AMVL production and yields during the cultivations of** ***A. oryzae*** **MTG4 using three different media**

	**Medium**		
	**Optimized complex medium**	**Optimized defined medium**	**MEB**
μ _max_ (h^−1^)	0.080 ± 0.002	0.052 ± 0.003	0.039 ± 0.001
Maximum titer of AMVL (mg/L)	250.9 ± 7.1	201.3 ± 13.9	58.8 ± 5.4
Y_xp_ (mg AMVL/g dw)	17.4 ± 0.5	20.8 ± 1.4	13.6 ± 1.3
r_p_ (mg AMVL/g dw/h)	1.02 ± 0.03	1.22 ± 0.08	0.62 ± 0.06

## Discussion

During the past decade, the technology of microbial secondary metabolite or bioactive compound production has undergone significant advances in the biotechnological and pharmaceutical industries. Recombinant products in particular have drawn much interest due to their great bioactivity and increasing applications. This includes the substances produced by non-native producers or heterologous products. Many efforts have been made regarding the efficient production of these compounds in well-characterized and fast-growing hosts using medium optimization and strain re-engineering to achieve the maximum yields. In this study, a recombinant strain, *A. oryzae* MTG4, was applied. This strain carries the gene encoding polyketide synthase from *Xylaria* sp. BCC 1067 and is capable of producing the product identified as a 2-pyrone containing compound, known as AMVL. The present work is the first report to assess medium components for the efficient heterologous production of AMVL.

Carbon and nitrogen sources are crucial factors affecting AMVL production. According to the results of the screening of carbon and nitrogen sources, the strain was able to utilize all carbon sources, including glucose, sucrose and soluble starch, and all nitrogen sources, including NH_4_^+^, NO_3_^−^, and yeast and malt extracts, suggesting that the recombinant *A. oryzae* MTG4 can utilize a broad spectrum of nutrients.

Several reports have illustrated the use of sucrose instead of glucose for the growth of microorganisms and secondary metabolite production. Glucose is found to be the strongest catabolite repressor, and many examples of secondary metabolites have been reported to be repressed by the presence of glucose in the cultivation media (Ruijter and Visser [1997]; Jonsbu et al. [2002]). Soluble starch had also been used as a carbon source in various types of microorganisms. It had been reported that enzyme synthesis in *Aspergillus terreus* and *Trichoderma viride* was induced in the presence of soluble starch (Ghosh et al. [1990]; Schellart et al. [1976]). Additionally, the majority of *Streptomyces* prefer starch as a carbon source for the production of secondary metabolites (Jia et al. [2008]; Gao et al. [2009]). Although, there are no observations of soluble starch acting as a repressor, soluble starch was not a suitable carbon source for AMVL production by *A. oryzae* MTG4 due to the low amount of amylase produced by this strain; thus, this strain cannot efficiently utilize starch as a substrate (Punya et al., [2013]; Kalayanamitr et al. [1987]).

Sucrose is the predominant sugar produced from sugarcane, which is one of the largest crops in Thailand. Because it is relatively cheap and abundant, and a higher product yield was observed in the media containing sucrose, sucrose is considered to be the optimal carbon source for the cultivation of *A. oryzae* MTG4. Several studies have shown that sucrose was the main nutritional factor for the production of secondary metabolites in fungi. The amount of sucrose used in the media varied depending on the strain. Only 3 g/L of sucrose was used as the main carbon source in Tannic acid-nitrate broth for the cultivation of *Fusarium*. To support growth and metabolite production by *Aspergillus* and *Penicillium*, 40 g/L of sucrose was supplemented in Czapek yeast extract broth and 150 g/L in yeast extract sucrose broth (YES). However, the concentration of sucrose could be as high as 200 g/L or 400 g/L in CZ20S (Czapek sucrose broth) and M40Y (Malt yeast broth), respectively, for the cultivation of xerophilic fungi such as *Eurotium* and *Wallemia* (Samson and Pitt, [1985]; Samson et al. [2004]).

Yeast extract and NaNO_3_ were considered as efficient nitrogen sources in this study. Several studies reported the use of yeast extract and NaNO_3_ for metabolite production in fungi (Barratt et al. [1965]; Frisvad [1981]; Klich and Pitt [1988]; Filtenborg et al. [1990]; Srinubabu et al. [2007]). Previous work suggested that NO_3_^−^ was superior to NH_4_^+^ as a nitrogen source for the biomass and the production of extracellular enzymes in *A. oryzae* (Kundu et al. [1973]). These results contradict the findings of Pedersen and Nielsen ([2000]), who reported that the a mixture of NH_4_^+^ and complex nitrogen sources, such as yeast extract, gave better biomass yield and enzyme productivity for *A. oryzae* than NH_4_^+^ or NO_3_^−^ as the sole nitrogen source. However, these experiments were carried out in chemostat cultures with pH control. Carlsen et al. ([1996]) showed that when *A. oryzae* was cultivated using NH_4_^+^ as the sole nitrogen source with no pH control, ammonium was taken up with a proton, resulting in a substantial acidification of the medium. The acidification had a negative effect on growth and led to the inactivation of enzyme production in *A. oryzae*, but this did not happen when NO_3_^−^ was used as a nitrogen source. This result indicates that the carbon and nitrogen sources used in the cultivation media completely depended on the strain and the cultivation process.

After sucrose, yeast extract and NaNO_3_ were identified as the important carbon, organic nitrogen and inorganic nitrogen sources, respectively, other components such as KH_2_PO_4_, K_2_HPO_4_, KCl, MgSO_4_.7H_2_O, CaCl_2_.2H_2_O, Na_3_C_6_H_5_O_7_.2H_2_O, biotin and trace elements were investigated by the PBD to determine whether these factors have significant effects on AMVL production. Because the use of yeast extract in the complex medium was not economical, the production of AMVL in the optimal defined medium without yeast extract was established for a comparison. The advantage of using the defined medium is that this medium produces more consistent titers, which allows easier process control and simplifies downstream recovery of AMVL.

KH_2_PO_4_ and K_2_HPO_4_ were used in the media because they act as buffering agents and provide the phosphate necessary for the production of sugar phosphates, nucleic acids, ATP and membrane phospholipids (Deacon [2006]). Sodium citrate or Na_3_C_6_H_5_O_7_.2H_2_O can also act as buffering agents or acidity regulators, resisting changes in pH. KCl was added to maintain the osmotic equilibrium. The inorganic salt, MgSO_4_.7H_2_O, stimulates fungal growth and enhances sporulation. CaCl_2_.2H_2_O provides Ca^2+^, which induces a loose pelleted form of growth and plays an important role in signal transduction in fungi (Pera and Callieri [1999]; Miller et al. [1990]). Biotin is a cofactor for acetyl-CoA carboxylase, which catalyzes the carboxylation of acetyl-CoA to form malonyl-CoA and is required for the de novo biosynthesis of long-chain fatty acids and polyketide biosynthesis (Hasslacher et al. [1993]; Wattanachaisaereekul et al. [2008]). Trace elements supply the Zn^2+^, Cu^2+^, Mn^2+^, and Fe^2+^ required for the functional components of enzymes produced by fungi. Fe^2+^ is particularly important in cytochrome P450 oxidases, which are involved in the biosynthesis of secondary metabolites. Zn^2+^ plays a crucial role in stabilizing protein structures (Hanson [2008]).

For the complex medium, sucrose, NaNO_3_, yeast extract and K_2_HPO_4_ were selected by the PBD as the major factors that influence AMVL production, while NaNO_3_, K_2_HPO_4_, KH_2_PO_4_ and trace elements were the important components affecting AMVL production in the defined medium. The results from the CCD shown in Figures 3 and 4 revealed that the increase of carbon source increased AMVL production until the optimal point was reached. A further increase of the sucrose concentration reversed the trend, most likely because the very high sucrose concentration in the media caused hyperosmotic stress, which may be detrimental to growth, cell survival and secondary metabolism in various fungi (Hafnawy [2001]; Duran et al. [2010]).

The amount of the nitrogen source in the media has been shown to be a critical factor in metabolite production. In this study, it was found that increased yeast extract or NaNO_3_ resulted in increased AMVL production. However, AMVL production was decreased at high concentrations of nitrogen sources. Previous work reported that excess nitrogen sources had been implicated in the repression of secondary metabolism such as penicillin biosynthesis in *Penicillium* sp. (Sanchez et al. [1981]). For secondary metabolite production, the concentrations of both carbon and nitrogen sources and their balance, or the C:N ratio, are very important. According to the results, higher AMVL production was observed in the N-limited medium with the optimal molar C:N ratio in the defined medium of 22:1 (70 g/L sucrose and 9.69 g/L NaNO_3_). The result from this study is similar to the findings of Mao et al. ([2005]), who reported that the highest cordycepin production in *Cordyceps militaris* was attained in the culture with a high C:N ratio. Additionally, López et al. ([2003]) illustrated that the maximum productivity of lovastatin by *A. terreus* was obtained in the nitrogen-limited media or the media with a high C:N ratio. This could be because secondary metabolite production is generally associated with nitrogen-limited growth when excess carbon can be channeled into secondary metabolism (Demain [1986]).

It was not surprising that KH_2_PO_4_ and K_2_HPO_4_ were important nutrients in the defined media in this study because phosphate salts provide a buffering capacity against pH fluctuations that could adversely affect normal metabolic activity. Phosphate is also known to be important for attaining high biomass densities of various fungi. It is also the crucial growth-limiting nutrient in many secondary metabolite fermentations. Thus, supplementing the media with phosphate enhanced AMVL production in *A. oryzae* MTG4. However, AMVL production decreased when a high concentration of phosphate was applied. This result is consistent with the report of Demain ([1986]), who showed that phosphate in the range of 0.3-300 mM generally supports extensive cell growth, whereas concentrations of 10 mM and above suppress the biosynthesis of many secondary metabolites.

The optimized complex and defined media were subsequently validated and compared with the original malt extract broth or MEB. Three different media cultivations in Figure 5 illustrated that the strain grew at different growth rates. The high maximum specific growth rates (μ_max_) of *A. oryzae* MTG4 in the optimized media were accomplished. After the exponential phase, the biomass had a decreasing trend in all cultivations, but AMVL production continued to increase until 16–17 days. Because AMVL is synthesized using the *pksmt* gene, it is classified as a secondary metabolite. Although a constitutive *gpdA* promoter was used to control the gene, there was a delay in the production of AMVL because it requires the enzymatic reaction to convert the intermediates or end products of primary metabolism to AMVL. In batch cultivation, cultures that exhibit a distinct growth phase are often followed by the secondary metabolite production phase. However, the timing of the primary and secondary phase may overlap depending on the strain and the nutrients in the culture.

During the cultivation of *A. oryzae* MTG4, the pH of the optimized complex and the MEB media increased with fungal growth. In contrast, the pH of the defined medium during the exponential phase did not increase but was almost constant at 6.3-6.7. This result might be due to the buffering effect of KH_2_PO_4_ and K_2_HPO_4_ present in the defined media. After that, the pH of all cultures dramatically increased, especially in the defined medium. The pH was maintained at over 8.0 for MEB and the complex medium and at 9.0 for the defined medium after 10 days of cultivations. It has been reported that the pH requirements for the growth of fungi is broad, ranging from 2.5 to 9.0. Generally, *Penicillium* species are tolerant of acidic pH, while *Aspergillus* species appear to be more tolerant of alkaline pH (Wheeler et al. [1991]). However, previous reports have shown that the growth of fungi could be affected by the pH of the culture medium either directly by the action of pH on cell surfaces or indirectly by the effect of substrate availability (Ahmed and Naresh [2009]). In our case, the decrease in biomass, along with the increase in pH, was caused by the limiting of media components because sucrose was completely exhausted at approximately 6–7 days of cultivation.

Additionally, the pH level may affect secondary metabolite production by the regulation of the gene encoding PacC (Espeso et al. [1993]; Denison [2000]). Under alkaline conditions, PacC is a transcriptional activator of alkaline-expressed genes and a repressor of acid-expressed genes. Several genes in secondary metabolism have been identified as alkaline-expressed genes, such as the genes required for penicillin production in *Penicillium chrysogenum* and *A. nidulans*. Thus, when growth was at an alkaline pH, higher levels of penicillins were observed (Shah et al. [1991]; Espeso et al. [1993]). Other examples of alkaline-expressed genes in secondary metabolism include genes in sterigmatocystin biosynthesis in *A. nidulans* (Keller et al. [1997]). According to the results, a high AMVL production was observed with the optimized media, even though the pH of the media was high. Therefore, it is possible that genes involved in AMVL biosynthesis may be controlled by a pH regulatory system.

Although the AMVL yields and productivities from the optimized media were improved compared to the original medium, this experiment was a preliminary study that requires further studies for the improvement of production. Additional process parameters such as aeration and pH should be investigated, and physiological fermentation in a controlled bioreactor should be studied in detail. The present study certainly promotes the development of biotechnological applications of AMVL and provides a basis for future studies with large-scale fermentation for the production of AMVL and other novel fungal secondary metabolites using *A. oryzae* as a heterologous host.

## Competing interests

The authors declare that they have no conflict of interests.
